# The relationship between Problem Gambling Severity Index scores and suicidality: Results of a 9‐year cohort study of young United Kingdom adults

**DOI:** 10.1111/add.70156

**Published:** 2025-09-16

**Authors:** Oliver Bastiani, Jasmine Khouja, Anya Skatova, Philip Newall

**Affiliations:** ^1^ School of Psychological Science University of Bristol Bristol UK; ^2^ Medical Research Council Integrative Epidemiology Unit Bristol Medical School, University of Bristol Bristol UK; ^3^ Population Health Sciences, Bristol Medical School University of Bristol Bristol UK

**Keywords:** ALSPAC, gambling‐related harm, longitudinal, PGSI, suicidality, suicide attempts

## Abstract

**Background and Aims:**

Previous studies report cross‐sectional associations between harmful gambling and suicidality. Longitudinal evidence is less common, but among young adults in the United Kingdom (UK), current longitudinal evidence highlights the specific association between increases in harmful gambling and subsequent suicidality. In a young UK adult cohort, we aimed to investigate whether harmful gambling, as measured by the Problem Gambling Severity Index (PGSI), would be associated with concurrent and future suicide attempts (at intervals of one, four and five years). Furthermore, the four‐year window was used to observe whether PGSI increases were a unique risk factor for suicidality, as has been observed before. In all instances, hierarchical logistic regression models explored whether associations were robust to controls for adolescent suicidality and relevant confounders.

**Design:**

A birth cohort study of the UK general population.

**Setting:**

Avon, UK.

**Participants:**

*n* = 2801 (62.4% female) participants with data for suicidality at 24 years from the Avon Longitudinal Study of Parents and Children (ALSPAC).

**Measurements:**

Past‐year non‐fatal suicide attempt prevalence was self‐reported at 24 and 25 years (in 2017–2018). PGSI was measured at 20 and 24 years. Measured confounders were sex, maternal education, economic activity (employment/education status), hyperactivity, alcohol disorder likelihood and adolescent suicidality.

**Findings:**

Past‐year suicide attempt prevalence was 2.57% at 24 years, and 1.86% at 25 years. Confounder‐adjusted models found that PGSI predicted suicide attempts at 24 years [odds ratio (OR) = 1.13, 95% confidence interval (CI) = 1.05–1.21, *P* = 0.001], and predicted future suicide attempts over one‐year (OR = 1.15, 95% CI = 1.06–1.25, *P* = 0.001) and four‐year timespans (OR = 1.20, 95% CI = 1.08–1.34, *P* < 0.001), but there was inconclusive evidence of this association over five years (OR = 1.14, 95% CI = 0.995–1.31, *P* = 0.058). There was inconclusive evidence that increases in PGSI scores between 20 and 24 years predict suicide attempts at 24 years (OR = 1.69, 95% CI = 0.72–3.99, *P* = 0.230).

**Conclusions:**

Among young adults in the United Kingdom, harmful gambling appears to be associated with suicide attempts; however, this association may be more complex long‐term, and increases in harmful gambling during adulthood may not be an important factor.

## INTRODUCTION

Gambling policy is a topic with international relevance, with the United Kingdom (UK) recently increasing restrictions on its regulated market [1], and the United States (US) contrastingly opening new legal sports betting markets [2]. The United Kingdom recently proposed to bring in a lower online–slots stake‐limit for 18‐ to 24‐year olds (£2) than older adults (£5) [3], in recognition of the elevated gambling harms among young adults [4], but these changes have not been implemented at the time of writing. Any potential restrictions on gambling's legal availability might be supported by evidence on harms felt by high‐risk gamblers [5], the larger group of at‐risk gamblers [6] and by affected others [7]. Gambling‐related death from suicide is an extreme harm, which formed a substantial proportion of the estimated burden of gambling‐related harm in an influential Public Health England report [8]. These findings were strongly contested by the gambling industry, who argued, for example, that the report inappropriately inferred population‐based harm from the most at‐risk cohort [9]. However, the UK government also highlighted that people who gamble are at risk of suicide in its recent suicide prevention strategy and called for more research on gambling‐related suicidality [10]. Research should, therefore, continue to explore associations between gambling and suicidality, using a range of measures, sample populations and timescales. This will allow policymakers to make the best estimates of the harms versus benefits of gambling and set policies accordingly.

A meta‐analysis of cross‐sectional evidence suggests that individuals with gambling disorders are more likely to report lifetime suicidal ideation and non‐lethal suicide attempts compared to others [11]. Suicide is further believed to account for between 5% and 31% of mortality among clinical gambling populations [12, 13]. Clinical cohort studies can lead to many insights on this most at‐risk cohort [13], but can make it hard to generalise effects to broader populations [9].

Population‐based studies that include non‐clinical populations are useful to investigate the association between gambling and suicidality among broader populations, especially among young adults, who are at higher risk of experiencing gambling‐related harm [4]. In one UK‐based cross‐sectional study, higher Problem Gambling Severity Index (‘PGSI’) [14] scores were associated with past‐year suicide attempts in a sample of 3549 young adults age 16 to 24 years [Mean (*M*) = 19.9], even when controlling for relevant confounders such as alcohol use and impulsivity [15]. This cohort has been followed longitudinally, with a group of 1941 providing valid responses 1 year later [16]. This study's primary reported outcome was that between‐wave changes in PGSI were associated with later rates of non‐fatal suicide attempts (*P* = 0.037) when controlling for relevant confounders. The effect was concentrated among those whose PGSI scores increased (*n* = 133; OR = 2.74). However, in the only reported analysis associating overall initial PGSI scores with later suicidality (adjusted for relevant confounders), initial PGSI score did not predict later suicide attempts (*P* = 0.23). Such longitudinal research is valuable because it explores the potential directionality of this association, which is not possible in cross‐sectional studies.

Although this study [16] is foundational for the longitudinal study of how PGSI scores predict later suicidality among young adults, it has limitations. These longitudinal associations were only measured over 1 year, so longer timescales are worth considering. Furthermore, the initial measure of suicidality was taken at an age where the average participant had been able to gamble legally for almost 2 years. Reverse causality could, therefore, be an issue, with previous engagement with legal adult gambling opportunities potentially influencing this initial measure of suicidality, and thereby pushing the overall longitudinal association toward insignificance. Finally, the finding regarding between‐wave changes in PGSI scores had a large estimated OR, but occurred in only a small subset of data, which suggests that further replication attempts would be informative.

The present research aimed to address these issues by investigating all potential longitudinal associations between suicidality and PGSI among young people in a large United Kingdom birth cohort, the Avon Longitudinal Study of Parents and Children (ALSPAC). We tested whether PGSI and suicidality were associated cross‐sectionally at age 24 and associated longitudinally at intervals of 1‐ (ages 24–25), 4‐ (ages 20–24) and 5‐years (ages 20–25). We also replicated previous analyses [16] on whether between‐wave changes in PGSI (from ages 20–24) were associated with later suicidality at age 24. The ALSPAC dataset spans 34 years, meaning it is possible to adjust for suicidality up to 5 years before some of the first measures of PGSI. Adjusting for previous suicidality could more robustly assess whether the previous association found was because of reverse causation (i.e. prior suicidality influencing PGSI scores).

## METHODS

Ethical approval for the study was obtained from the ALSPAC Ethics and Law Committee, and the local research ethics committees. Informed consent for the use of data collected via questionnaires and clinics was obtained from participants following the recommendations of the ALSPAC Ethics and Law Committee at the time. Analyses were performed in STATA v.18, and all code is available online (https://github.com/oliverbastiani/ALPSAC-Gambling-Suicide-2024).

### Participants

**FIGURE 1:**
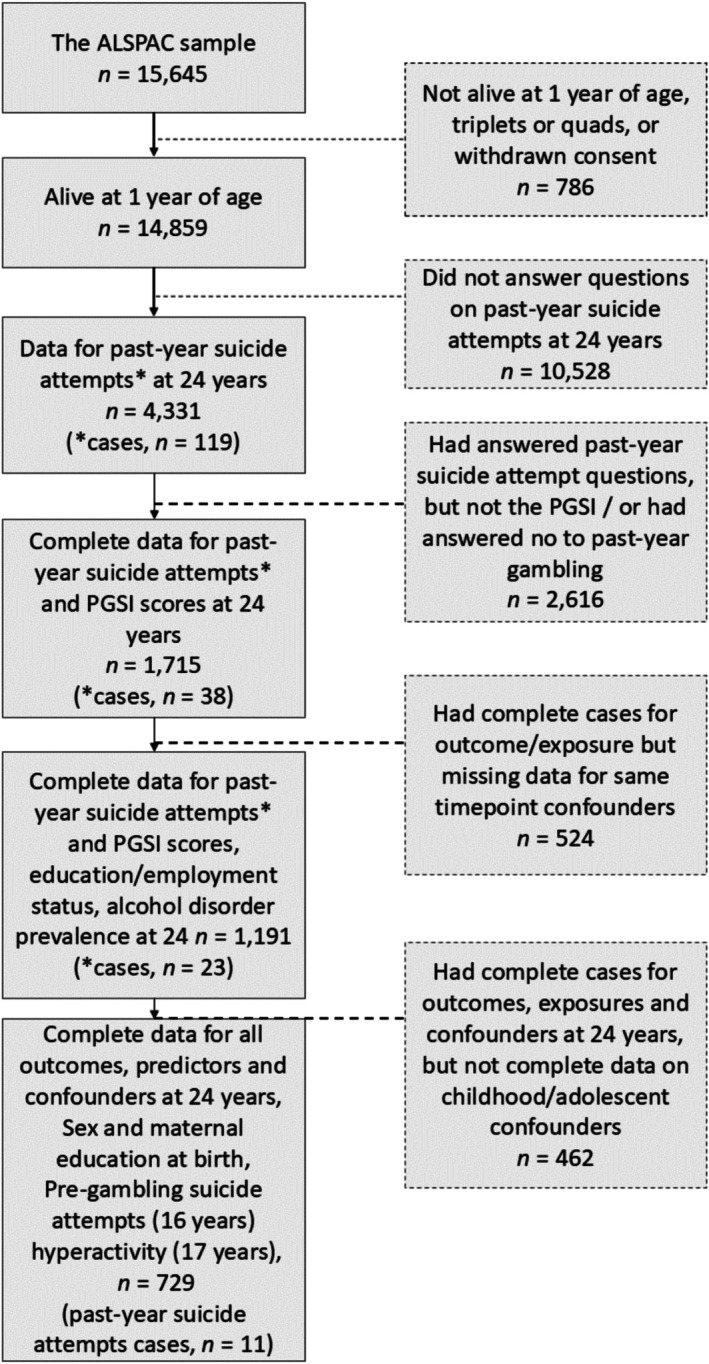
Flow diagram depicting sample sizes and attrition in the Avon Longitudinal Study of Parents and Children (ALSPAC) cohort, with data availability and past‐year suicide attempt case prevalence as variables that are added to complete case analysis. The Problem Gambling Severity Index (PGSI) is a 9‐item measure of harmful gambling for use in the general population.

Data were sourced from ALSPAC, a multi‐generational longitudinal cohort study that tracks health and lifestyle outcomes across a child's development [17, 18, 19]. Pregnant women resident in Avon, United Kingdom, with expected delivery dates between 1 April 1991 and 31 December 1992, were invited to take part in the study. The initial number of pregnancies enrolled was 14 541 and 13 988 children who were alive at 1‐year of age. After three additional phases of enrolment, the total sample size for analyses using any data collected after the age of 7 is, therefore, 15 447 pregnancies, and 14 901 children were alive at 1‐year of age. Overall, 14 203 unique mothers were initially enrolled in the study, which increased to 14 833 unique women (G0 mothers) after additional recruitment. A total of 12 113 G0 partners have been in contact with the study, and 3807 G0 partners are currently enrolled. The current study included all G1 children with complete‐case data for past‐year suicide attempts at 24 years. Study data were collected and managed using Research Electronic Data Capture (REDCap) electronic data capture tools hosted at the University of Bristol [20]. REDCap is a secure, web‐based software platform designed to support data capture for research studies. Questionnaire completion rates differed between waves and were subject to attrition with age, therefore, sample sizes differed between analyses (Figure 1). For full ALSPAC sample characteristics, please see Table 1. Please note that the study website contains details of all the data that is available through a fully searchable data dictionary and variable search tool (http://www.bristol.ac.uk/alspac/researchers/our-data/).

**TABLE 1:** Distribution of sample characteristics between subsamples.

	Original sample, *n* = 15 645 (%)	Sample with complete‐case data for suicide attempts at 24 years, *n* = 4331 (%)	Sample for fully adjusted complete case analysis, *n* = 729 (%)	Imputed sample for fully adjusted analysis, *n* = 2801 (%)
Child's sex—male (ref: female)	7684 (51.1)	1508 (34.8)	324 (44.4)	1055 (37.6)
Parental social class (ref: non‐manual)	2245 (19.4)	452 (12.2)	58 (8.2)	400 (14.3)
Maternal education (ref: A‐level or greater)	8071 (64.7)	2045 (52.8)	335 (46.0)	1607 (57.4)
Home ownership status (ref: mortgaged/owned)	3615 (26.8)	597 (15.4)	79 (11.0)	471 (16.8)
Past‐year suicide attempts at 25 years (ref: 0 past‐year attempts)	66 (1.62)	52 (1.55)	7 (1.12)	52 (1.86)
Past‐year suicide attempts at 24 years (ref: 0 past‐year attempts)	119 (2.75)	119 (2.75)	11 (1.51)	72 (2.57)
Lifetime suicide attempts at 16 years (ref: 0 past‐year attempts)	296 (5.78)	177 (5.77)	28 (3.84)	162 (5.79)

### Measures

#### Suicidality

**FIGURE 2:**
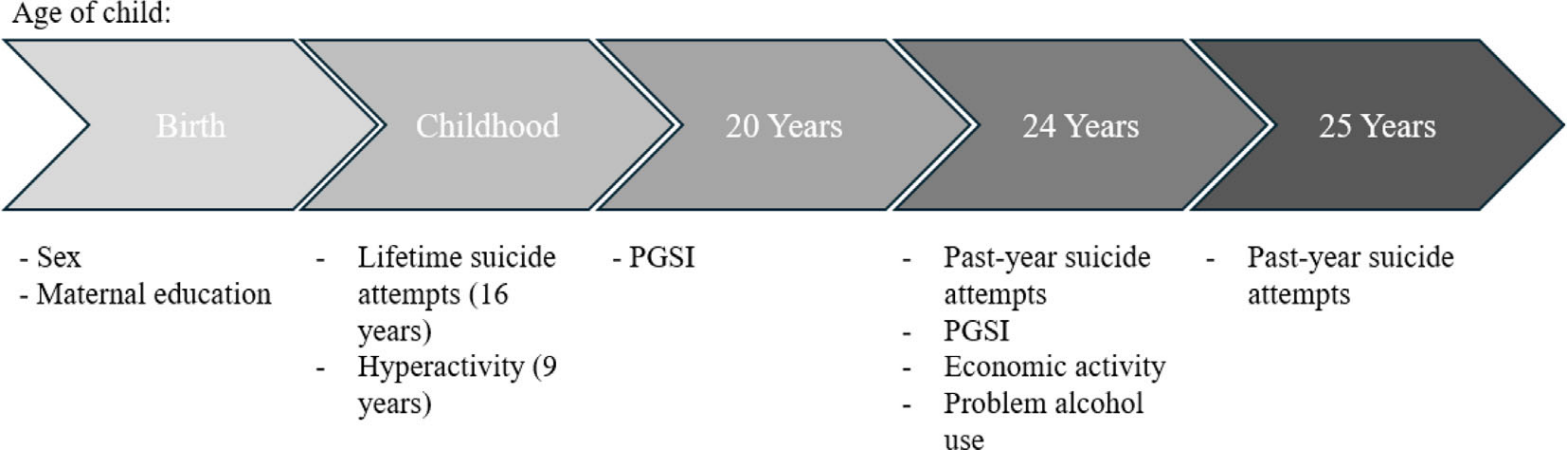
A timeline depicting the ages at which each variable used in our analyses were collected from the study participants. The Problem Gambling Severity Index (PGSI) is a 9‐item measure of harmful gambling for use in the general population.

The dataset included self‐completed measures for non‐lethal suicide attempts at ages 16 (collected between October 2007–August 2009), 24 (collected between November 2016–August 2017) and 25 years (collected between November 2017–July 2018). As certain survey questions were dependent on earlier survey responses, we have, at ages 16 and 24, combined responses to related measures to create more inclusive measures of suicidality. All measures of suicidality were coded as binary outcome variables (yes/no). See Table S1 for the full survey questions and responses, and Figure 2 for a visual timeline depicting the ages at which each variable was collected.

At age 16, participants who had previously responded ‘yes’ to, ‘Have you ever hurt yourself on purpose in any way’ were asked, ‘On any of the occasions when you have hurt yourself on purpose, have you ever seriously wanted to kill yourself?’ Furthermore, participants who had previously responded with ‘yes’ to ‘Have you ever hurt yourself on purpose in any way (e.g. by taking an overdose of pills or by cutting yourself)?’, were asked, ‘Do any of the following reasons help to explain why you hurt yourself on that occasion?’ (referring to the last time they had hurt themselves on purpose). Non‐fatal suicide attempts were indicated if participants responded ‘yes’ to the first question or responded, ‘I wanted to die’ to the second question. These responses to both questions were pooled to create the most inclusive measure of lifetime suicide attempts at 16 years, following the convention of previous ALSPAC studies [21].

At age 24, all participants were asked, ‘Have any of these happened since you were 23 years old and did they affect you? – You attempted suicide’. Furthermore, participants who had previously responded with ‘yes’ to ‘Have you ever hurt yourself on purpose in any way (e.g. by taking an overdose of pills or by cutting yourself)?’ were asked, ‘when was the last time you hurt yourself on purpose and you seriously wanted to kill yourself?’ Non‐fatal suicide attempts were indicated if participants responded ‘yes’ (with any degree of affect) to the first question, or responded ‘in the last week’ or, ‘more than a week ago, but in the last year’ to the second question. These responses to both questions were pooled to create the most inclusive measure of past‐year suicide attempts at 24 years.

At age 25, all participants were asked, ‘Have any of these happened in the past 12 months and did they affect you? – You attempted suicide’. Non‐fatal past‐year suicide attempts at 25 years were indicated if participants responded ‘yes’ (with any degree of affect) to this question.

#### Harmful gambling

Harmful gambling was measured with the PGSI, the gold standard nine‐item questionnaire used to assess harmful gambling in the general population [14, 22, 23]. Each question has four response options ranging from ‘never’, to ‘almost always’, which are scored from 0 to 3, respectively. ALSPAC participants completed the PGSI at ages 20 and 24 years, in the same questionnaire session as the suicidality/self‐harm questions. The distributions of PGSI scores at both ages and the score changes between these are shown in Table 2.

**TABLE 2:** Distributions of PGSI scores at ages 20 and 24 years, in the complete‐case and imputed ALSPAC samples.

	Complete‐case sample		Imputed sample	
	Age		Age	
	20, *n* = 2622	24, *n* = 1716	20, *n* = 2801	24, *n* = 2801
PGSI scores				
0	1866 (71.2%)	1275 (74.3%)	1971 (70.4%)	2144 (76.5%)
1–2	446 (22.4%)	321 (18.7%)	654 (23.3%)	496 (17.7%)
3–7	144 (5.49%)	90 (5.2%)	152 (5.41%)	126 (4.49%)
8+	25 (0.95%)	30 (1.75%)	24 (0.86%)	35 (1.25%)
Change in PGSI scores:		20–24 (*n* = 893)		20–24 (*n* = 2801)
0 (no change)	N/A	567 (63.5%)	N/A	1832 (65.4%)
Increase (1 or more PGSI point)	N/A	134 (15.0%)	N/A	410 (14.6%)
Decrease (1 or more PGSI point)	N/A	192 (21.5%)	N/A	559 (20.0%)

Abbreviations: ALSPAC, Avon Longitudinal Study of Parents and Children; PGSI, Problem Gambling Severity Index.

#### Confounding variables

Confounding variables with known associations with suicidality or harmful gambling were selected from the ALSPAC dataset. Consistent with Wardle *et al*.’s methods [16], confounders were parsimoniously chosen if they were associated with past‐year suicide attempts at 24 or 25 years in preliminary univariate logistic regression models (Tables S2 and S3). Our final analyses included the following confounders as covariates: sex, maternal education, economic activity (employment/education status), hyperactivity, alcohol disorder likelihood and age‐16 suicidality. The two covariates that act as common proxies of socio‐economic status (SES) were parental‐completed maternal education level (measured between March 1991 and January 1993) [24, 25] and participant‐completed economic activity at 24 years (clinic‐reported between June 2015 and September 2017). Economic activity measured whether participants were in either part‐ or full‐time education or employment or in a training scheme and were categorized as being economically active if they reported yes to any of these (vs. no). Hyperactivity was measured with the hyperactivity subscale of the Strengths and Difficulties Questionnaire [26], at 9.5 years (parent‐reported between February 2001 and November 2002), and was chosen as the closest proxy for impulsivity in ALSPAC. Conduct problems, locus of control and diagnoses of attention deficit hyperactivity disorder (ADHD) were also considered as impulsivity proxies, but hyperactivity had the strongest associations with suicidality and was included over the other proxies because of potential multicollinearity issues. Alcohol disorders were measured with the Alcohol Use Disorders Identification Test Short Form (AUDIT‐C) [27] and were administered by clinicians during attendance of a clinic at 24 years of age (between June 2015 and September 2017). Self‐reported lifetime suicide attempt prevalence at 16 years was included as an early measure of suicidality, as heavy gambling on age‐restricted gambling formats is rare during adolescence [28].

Other potential confounding variables were considered. Cigarette smoking status at 24 years was considered, but was ruled out during preliminary univariate analyses because of a lack of association with suicide attempts at ages 24 and 25 years. Illicit drug use was considered as a potential confounder, but at 24 years this was only measured with lifetime or past‐year use of specific drug types, rather than a validated questionnaire of illicit drug harms. Therefore, we excluded this measure in favour of the AUDIT for alcohol use disorders, as a reflection of risk‐taking behaviours at this age. Mental health measures, such as depression and anxiety, were considered, but these were filtered out because of their demonstrated associations with measures already included in the model: AUDIT scores [29] and suicidal self‐harm at 16 years [30]. For the full descriptions of considered confounders, their respective measurements and their dates of data collection, please see Table S1.

### Statistical analyses

These analyses were not pre‐registered and should be considered exploratory.

#### Analyses on overall PGSI scores

Bivariate logistic regression analyses were initially conducted, with each PGSI measure as the predictor, and past‐year suicide attempt prevalence at ages 24 and 25 years as the outcomes (model 1a‐4a). Multi‐variate logistic regression models were then run hierarchically in three additional steps, adjusted with the parsimoniously chosen covariates mentioned above. These three steps added: socio‐demographic factors (sex, maternal education, economic activity; model 1b‐4b, 5c), behavioural factors (hyperactivity, alcohol disorder likelihood; model 1c‐4c, 5d) and age‐16 suicidality; model 1d‐4d, 5e. Hierarchical regression models (sequential models) added predictors in researcher‐specified groupings, to demonstrate how additional covariates affect the importance of the independent variables retained from previous models [31]. The inclusion of age‐16 suicidality in the final step served as the most robust test of whether these associations might be because of a causal effect of gambling or instead reflect an instance of reverse causation (e.g. where harmful gambling might be used as an escape from feelings of suicidality). For congruence with Wardle *et al*.,’s prior research [15, 16], we did not differentiate between non‐gamblers and gamblers with a PGSI score of 0, as the investigation focused on the effect of harmful gambling, rather than gambling prevalence/frequency.

First, cross‐sectional analyses were run using data from age 24 to see if PGSI scores would predict past‐year suicide attempts. Second, the three potential longitudinal analyses were run, to see if PGSI scores at age 20 could predict past‐year suicide attempts at ages 24 and 25 and whether PGSI scores at age 24 would predict past‐year suicide attempts at age 25.

#### Analyses of PGSI score changes

For congruence with Wardle *et al*.,’s prior longitudinal research [16], the associative analysis between suicidality and PGSI score changes that most closely mirrored theirs was also explored. A univariate logistic regression model was run with the categorical change (categorised as no change/increase by 1 or more PGSI point/decrease by 1 or more PGSI point) in PGSI scores between 20 and 24 years as the predictor, and past‐year suicide attempts at 24 years as the outcome (model 5a). Wardle *et al*., [16] reported specifications with and without controls for overall initial PGSI scores, but the main reported analysis was the one with this additional control variable. Overall initial PGSI scores were, therefore, added in the first multi‐variate logistic regression model run here (model 5b), and with the other confounder variables then added in the same sequence as before.

#### Imputation to address data missingness

As standard in ALSPAC cohort research [32, 33, 34], multiple‐chained imputation was conducted on missing data (see Figure 1). This was necessary here, as cases of suicide attempts attenuated greatly because of missingness in fully adjusted models: with 119 reported cases at 24 years reduced to as low as seven cases in some fully adjusted analyses (Tables S4–S8). We used the ‘mi impute chained’ command in STATA to conduct multiple imputations with the chained equations method (MICE) [35]. The MICE method generates multiple different imputed ‘complete’ datasets, whereby missing data were imputed by considering the observed values for each participant and the relations between variables that were observed among other participants [36]. We used MICE to generate 50 imputed datasets, such that there were 50 predictions for each missing variable. These 50 datasets were pooled via Rubin's rules [37], such that each imputed value reflected averages across these 50 imputations. This method decreases any uncertainty caused by individual imputations.

This model assumes that data are missing at random. The probability of a variable being missing is associated with the values of other observed variables, but not the missingness of other variables. Cases were included in the imputation model dependent on whether participants had complete data for past‐year suicide attempts at 24 years and had answered ‘yes’ to past‐year gambling at 24 years, such that PGSI scores were generated appropriately, resulting in an imputed sample of *n* = 2801. Estimates were obtained by pooling results across 50 imputed datasets via Rubin's rules [37]. Proportions of values for each imputed variable were compared against their respective complete cases to check that imputations were representative of their original variables. Additionally, all main analyses were repeated using the complete‐case (non‐imputed) subsample of data to investigate whether our use of imputation had driven any observed effects.

Demographically, the imputed sample for fully adjusted analysis was similar to the sample with complete‐case data for suicide attempts at 24 years, and both samples had a greater proportion of women and people with higher SES than the original ALSPAC sample (Table 1). The imputed sample for fully adjusted analysis contained higher percentages of people with low SES and suicide attempts compared to the sample for fully adjusted complete case analysis. This is likely because of variable missingness in the sample for fully adjusted complete case analysis as its demographic distributions were further from the original sample's than the imputed sample for fully adjusted analysis. For the number and percentages of data that were imputed for each variable, please see Table S9.

## RESULTS

The imputed sample contained 2801 participants. In the complete‐case ALSPAC sample, high‐risk gambling prevalence (PGSI scores 8+) was 0.95% at 20 years and 1.75% at 24 years. The majority of participants who completed both PGSI questionnaires had no change in PGSI scores between 20 and 24 years, but a greater proportion of people reduced their PGSI score by at least one point (*n* = 192, 21.5%), than increased it (*n* = 134, 15.0%). PGSI scores were more stable between 20 and 24 years for women (70.1%) compared to men (55.1%), and a greater proportion of men (19.1%) than women (11.8%) increased their PGSI score by at least 1 point [χ^2^ test: χ^2^(2, *n* = 893) = 21.59, *P* < 0.001]. For the proportions of PGSI scores and score changes between 20 and 24 years, among the complete‐case and imputed samples, please see Table 2. Past‐year suicide‐attempt prevalence was 2.75% of the full sample at 24 years and 1.62% at 25 years. Lifetime suicide‐attempt prevalence was 5.78% at 16 years, 5.78% at 20 years and 7.43% at 24 years. All analyses of the associations between suicide attempts and PGSI scores and PGSI score changes were also run with the complete‐case (unimputed samples). The results of these complete‐case analyses are available in Tables S4–S8. The results of the analyses restricted to complete‐case subsamples were similar, but less statistically powered, compared to the results of the imputed sample. In brief, the complete‐case subsample analyses showed moderate evidence for longitudinal relationships across 1‐, and 4‐year horizons, but not cross‐sectionally at 24 years.

### The association between PGSI scores and suicidality

The results of hierarchical adjustments of confounding variables in the overall PGSI score analyses can be found in Tables 3, 4, 5, 6. Table 3 shows that there was a consistent cross‐sectional positive association between PGSI and suicidality at age 24 (bivariate model: OR = 1.13, 95% CI = 1.07–1.21, *P* < 0.001; fully adjusted model: OR = 1.13, 95% CI = 1.05–1.21, *P* = 0.001). A cross‐sectional association was, therefore, supported. Table 4 shows that there was consistent evidence of a 1‐year longitudinal positive association between PGSI scores at 24 years and past‐year suicide attempts at 25 years (bivariate model: OR = 1.17, 95% CI = 1.09–1.26, *P* < 0.001; fully adjusted model: OR = 1.15, 95% CI = 1.06–1.25, *P* = 0.001). Table 5 shows that there was consistent evidence of a 4‐year positive association between PGSI scores at 20 years and past‐year suicide attempts at 24 years (bivariate model: OR = 1.19, 95% CI = 1.09–1.30, *P* < 0.001; fully adjusted model: OR = 1.20, 95% CI = 1.08–1.34, *P* < 0.001). However, Table 6 shows that the evidence of associations was inconclusive between PGSI scores at 20 years and past‐year suicide attempts at 25 years in the bivariate model (OR = 1.15, 95% CI = 1.02–1.30, *P* = 0.027 and particularly in the fully adjusted model (OR = 1.14, 95% CI = 1.00–1.31, *P* = 0.058). The inconclusive evidence of this association was not solely because of the inclusion of age‐16 suicidality in the fully adjusted model, as the association was inconclusive in model 4b (OR = 1.15, 95% CI = 1.00–1.31, *P* = 0.050), although it was slightly stronger in model 4c (OR = 1.16, 95% CI = 1.01–1.32, *P* = 0.032). Longitudinal associations were, therefore, supported over 1‐ and 4‐year timeframes, but not consistently supported over 5‐year timeframes.

**TABLE 3:** Hierarchical adjustments of potential confounding variables for the association between past‐year suicide attempts at 24 years and PGSI scores at 24 years (*n* = 2801).

Variable	Model 1a (bivariate)		Model 1b		Model 1c		Model 1d (fully adjusted)	
	OR (95% CI)	*P*‐value	OR (95% CI)	*P*‐value	OR (95% CI)	*P*‐value	OR (95% CI)	*P*‐value
PGSI scores at 24 y	1.13 (1.07–1.21)	<0.001	1.34 (1.06–1.21)	<0.001	1.14 (1.07–1.21)	<0.001	1.13 (1.05–1.21)	0.001
Sex			1.95 (1.12–3.42)	0.019	2.07 (1.17–3.68)	0.013	1.47 (0.79–2.72)	0.224
Economic activity			0.22 (0.11–0.44)	<0.001	0.23 (0.11–0.47)	<0.001	0.25 (0.11–0.53)	<0.001
Maternal education			0.59 (0.33–1.08)	0.086	0.56 (0.31–1.03)	0.062	0.61 (0.33–1.14)	0.124
Hyperactivity					1.96 (0.89–4.34)	0.096	1.72 (0.71–4.15)	0.229
AUDIT scores					1.09 (0.96–1.24)	0.190	1.09 (0.96–1.24)	0.182
Lifetime suicide attempt prevalence at 16 y							9.04 (4.41–18.50)	<0.001

Abbreviations: AUDIT, Alcohol Use Disorders Identification Test; PGSI, Problem Gambling Severity Index.

**TABLE 4:** Hierarchical adjustments of potential confounding variables for the association between past‐year suicide attempts at 25 years and PGSI scores at 24 years (*n* = 2801).

Variable	Model 2a (bivariate)		Model 2b		Model 2c		Model 2d (fully adjusted)	
	OR (95% CI)	*P*‐value	OR (95% CI)	*P*‐value	OR (95% CI)	*P*‐value	OR (95% CI)	*P*‐value
PGSI scores at 24 y	1.17 (1.09–1.26)	<0.001	1.15 (1.07–1.24)	<0.001	1.16 (1.07–1.25)	<0.001	1.15 (1.06–1.25)	0.001
Sex			1.09 (0.51–2.31)	0.830	0.95 (0.44–2.04)	0.888	0.72 (0.31–1.67)	0.447
Economic activity			0.17 (0.07–0.39)	<0.001	0.19 (0.08–0.44)	<0.001	0.21 (0.09–0.49)	<0.001
Maternal education			0.53 (0.25–1.11)	0.094	0.60 (0.28–1.27)	0.181	0.64 (0.30–1.36)	0.245
Hyperactivity					1.82 (0.59–5.59)	0.294	1.67 (0.51–5.40)	0.392
AUDIT scores					0.85 (0.73–0.98)	0.023	0.85 (0.74–0.98)	0.029
Lifetime suicide attempt prevalence at 16 y							5.59 (1.88–16.66)	0.002

Abbreviations: AUDIT, Alcohol Use Disorders Identification Test; PGSI, Problem Gambling Severity Index.

**TABLE 5:** Hierarchical adjustments of potential confounding variables for the association between past‐year suicide attempts at 24 years and PGSI scores at 20 years (*n* = 2801).

Variable	Model 3a (bivariate)		Model 3b		Model 3c		Model 3d (fully adjusted)	
	OR (95% CI)	*P*‐value	OR (95% CI)	*P*‐value	OR (95% CI)	*P*‐value	OR (95% CI)	*P*‐value
PGSI scores at 24 y	1.19 (1.09–1.30)	<0.001	1.22 (1.11–1.32)	<0.001	1.21 (1.10–1.34)	<0.001	1.20 (1.09–1.34)	<0.001
Sex			2.06 (1.16–3.67)	0.013	2.16 (1.20–3.86)	0.010	1.54 (0.82–2.88)	0.178
Economic activity			0.20 (0.10–0.41)	<0.001	0.21 (0.10–0.44)	<0.001	0.23 (0.10–0.50)	<0.001
Maternal education			0.58 (0.32–1.05)	0.072	0.55 (0.30–1.01)	0.053	0.60 (0.32–1.13)	0.113
Hyperactivity					1.87 (0.84–4.14)	0.124	1.63 (0.67–3.99)	0.280
AUDIT scores					1.08 (0.95–1.22)	0.253	1.08 (0.95–1.23)	0.236
Lifetime suicide attempt prevalence at 16 y							9.08 (4.41–18.70)	<0.001

Abbreviations: AUDIT, Alcohol Use Disorders Identification Test; PGSI, Problem Gambling Severity Index.

**TABLE 6:** Hierarchical adjustments of potential confounding variables for the association between past‐year suicide attempts at 25 years and PGSI scores at 20 years (*n* = 2801).

Variable	Model 4a (bivariate)		Model 4b		Model 4c		Model 4d (fully adjusted)	
	OR (95% CI)	*P*‐value	OR (95% CI)	*P*‐value	OR (95% CI)	*P*‐value	OR (95% CI)	*P*‐value
PGSI scores at 24 y	1.15 (1.02–1.30)	0.027	1.15 (1.00–1.31)	0.050	1.16 (1.01–1.32)	0.032	1.14 (1.00–1.31)	0.058
Sex			1.01 (0.48–2.14)	0.981	0.88 (0.41–1.88)	0.732	0.67 (0.29–1.55)	0.347
Economic activity			0.16 (0.07–0.35)	<0.001	0.17 (0.07–0.40)	<0.001	0.19 (0.08–0.45)	<0.001
Maternal education			0.50 (0.24–1.03)	0.060	0.57 (0.27–1.19)	0.136	0.61 (0.29–1.28)	0.189
Hyperactivity					1.68 (0.55–5.16)	0.362	1.54 (0.47–4.97)	0.472
AUDIT scores					0.84 (0.73–0.97)	0.020	0.85 (0.73–0.98)	0.026
Lifetime suicide attempt prevalence at 16 y							5.66 (1.95–16.5)	0.002

Abbreviations: AUDIT, Alcohol Use Disorders Identification Test; PGSI, Problem Gambling Severity Index.

### The association between PGSI score changes and suicidality

The results of hierarchical adjustments of confounding variables in the PGSI score changes analyses can be found in Table 7. The bivariate logistic regression model revealed an association between PGSI score increases between 20 and 24 years and past‐year suicide attempts at 24 years (OR = 2.50, 95% CI = 1.17–5.36, *P* = 0.019). However, there was no clear evidence of this association in any of the other models, with ORs ranging from 1.69 to 2.08, and *P*‐values ranging from 0.077 to 0.230. These ORs are consistent with the possibility of an association, but the evidence is inconclusive because of the wide confidence intervals. By contrast, PGSI scores at 20 years consistently predicted suicidality at 24 years, with ORs ranging from 1.15 to 1.18 and *P*‐values ranging from 0.002 to 0.006, as are consistent with the associations observed between these variables in Table 5 above.

**TABLE 7:** Hierarchical adjustments of potential confounding variables for the association between past‐year suicide attempts at 24 years and categorical changes in PGSI scores from 20 to 24 years (*n* = 2801).

Variable	Model 5a (bivariate)		Model 5b		Model 5c		Model 5d		Model 5e (fully adjusted)	
	OR (95% CI)	*P*‐value	OR (95% CI)	*P*‐value	OR (95% CI)	*P*‐value	OR (95% CI)	*P*‐value	OR (95% CI)	*P*‐value
PGSI score change 20–24 years (categorical)										
PGSI score increased	2.50 (1.17–5.36)	0.019	1.89 (0.81–4.41)	0.077	1.89 (0.81–4.41)	0.140	1.85 (0.79–4.32)	0.155	1.69 (0.72–3.99)	0.230
PGSI score decreased	2.29 (1.00–5.26)	0.050	1.64 (0.68–3.97)	0.269	1.70 (0.69–4.20)	0.247	1.60 (0.42–3.98)	0.311	1.55 (0.60–3.98)	0.363
PGSI scores at 20 y			1.15 (1.05–1.26)	0.004	1.18 (1.06–1.30)	0.002	1.18 (1.06–1.30)	0.002	1.17 (1.05–1.31)	0.006
Sex					2.16 (1.22–3.84)	0.009	2.24 (1.25–4.02)	0.007	1.60 (0.85–2.99)	0.142
Economic activity					0.21 (0.10–0.44)	<0.001	0.21 (0.10–0.46)	<0.001	0.23 (0.10–0.52)	<0.001
Maternal education					0.59 (0.33–1.08)	0.087	0.57 (0.31–1.04)	0.065	0.62 (0.33–1.15)	0.129
Hyperactivity							1.80 (0.81–4.02)	0.152	1.58 (0.64–3.90)	0.315
AUDIT scores							1.07 (0.94–1.22)	0.289	1.08 (0.95–1.22)	0.268
Lifetime suicide attempt prevalence at 16 y									8.90 (4.30–18.4)	<0.001

Abbreviations: AUDIT, Alcohol Use Disorders Identification Test; PGSI, Problem Gambling Severity Index.

## DISCUSSION

Suicidality is a severe potential gambling‐related harm, but the extent to which harmful gambling is a risk‐factor for suicide has been questioned outside of clinical gambling populations [9]. Within the ALSPAC cohort, we observed associations between PGSI and suicidality, which were robust to the addition of covariates, both cross‐sectionally and longitudinally up to horizons of 4 years. There was inconclusive evidence of these associations when including covariates at a 5‐year horizon. Contrastingly, we only observed a clear bivariate association between PGSI increases and suicidality. These nuanced findings require further consideration in light of the previous literature [16].

Wardle *et al*.’s [16] study only reported a fully adjusted analysis for the relationship between PGSI scores at wave 1 and suicide attempts at wave 2. A model that excluded wave 1 suicide attempts was also conducted, but this model included the between‐wave change in PGSI scores, so we cannot determine the standalone association with past PGSI scores. Therefore, it is difficult to infer the possible impact of any concerns regarding potential reverse causality on longitudinal associations with PGSI scores and later suicide attempts, specifically. Their association between suicide attempts at wave 1 and suicide attempts at wave 2 had an OR of 6.27. This suggests that the reported analysis may largely reveal the tendency for suicidality to persist over horizons of a year. Wardle *et al*.’s [16] longitudinal analyses were also limited by focusing on PGSI increases, when PGSI scores only increased for a minority of that analytical sample (*n* = 133, 6.9%). In the present imputed sample, 14.6% increased their PGSI score between ages 20 and 24. This again resulted in a small number of participants with PGSI scores increasing between 20 and 24 years (*n* = 410), suggesting that much larger sample sizes will be required in future research to better understand the effect of longitudinal increases in PGSI scores. Overall, longitudinal analyses benefit from including people for whom gambling‐related harm is consistent, as well as increases, over time, as persistent gambling‐related harm is a common theme emerging from analysis of first‐person accounts [38, 39].

This conflicting pattern of results could add to the strength of previous evidence for gambling‐related harm being a risk‐factor for suicidality. Although the overall longitudinal analysis became inconclusive at the 5‐year horizon, the directionality and average size of estimated ORs were similar to the findings at shorter horizons. This suggests that issues such as statistical power, or the tendency for high‐risk gamblers to drop‐out of longitudinal surveys [40], may contribute to these results. Any potential association is likely to attenuate over longer time‐horizons, and even a potential causal effect limited to 4 years could have significant public health implications.

These results, therefore, have various policy implications. The latest official Great Britain statistics have noted a significant increase in estimated prevalence rates for harmful gambling [41], which could have implications for rates of future suicidality in the population. In January 2025, the National Institute for Health and Care Excellence announced new guidelines for identifying and treating gambling‐related harms, which highlight prior evidence on gambling and suicidality [42]. Furthermore, these results help add to the justification for the UK government's focus on gambling as a priority area in their suicide prevention strategy [10].

This study has various limitations. ALSPAC's findings are limited to a specific location and time. Although ALSPAC has had low rates of attrition compared to other cohort studies, its cohort has become less representative over time, with, compared to original enrolment demographics [17], more white, female individuals and fewer individuals from low‐income households. Different relationships could emerge over time, especially as new technologies have only increased gambling's presence in the United Kingdom, such as the recent growth of online gambling [43, 44] and gambling advertising on social media [45]. These new gambling technologies may produce different relationships with suicidality than those captured here, as our most recent measure of PGSI was measured in August 2017, and should be studied in future research. ALSPAC is a general‐purpose cohort, so gambling items were only asked inconsistently. There is, therefore, still a greater need for longitudinal data in gambling research, particularly those with objective measures of gambling activity [46].

Our selection of covariates was parsimonious. Other studies with greater power should include additional covariates such as depression, anxiety and illicit drug use to investigate whether these influence the relationship between PGSI and suicidality. Some suicide questions at ages 16 and 24 years were asked within a wider self‐harm questionnaire where participants could only answer suicidality‐related questions if they had already answered ‘yes’ to ever having self‐harmed. Some questions specifically measured suicide in a self‐harm context, asking if suicide was a reason for self‐harm, rather than a standalone behaviour. Therefore, variables may not have been sensitive enough to capture all past‐year suicidality occurrences, but these have been used in other fields to measure suicidality within ALSPAC [21]. Although the use of age‐16 suicidality reduced potential concerns around reverse causality, some children do gamble on age‐restricted products at this age [47], but no gambling‐related measures were collected in that ALSPAC wave. Additionally, our sample may suffer from survivor bias, as we cannot account for the responses of people who may have died by suicide. Other studies, which implement ‘psychological postmortems’ of gambling‐related suicides, may be valuable to triangulate with our evidence and address this potential bias [48, 49]. Furthermore, our results were only associative, so relevant causal methodologies and/or methodological triangulations are needed to better understand potential causal patterns.

Some limitations could occur from imputing missing data. We used the MICE model [35], which assumes that data are missing at random, but to the best of our knowledge there are no conclusive tests for this. It is plausible that the missingness of some variables may correlate with other variables' missingness. For example, suicide attempts at 16 years could make it less likely that participants complete questionnaires at later dates. However, our use of MICE is supported by our analyses of the complete‐case sample (Tables S4–S8). The complete‐case analyses results present similar associative trends to those of the imputed samples, with weak evidence of long‐term associations between suicide attempts and PGSI scores at 1‐ and 4‐year timeframes. This suggests that the imputed data does not differ largely from a complete‐case analysis. Future longitudinal studies focused on gambling‐related suicidality should prioritise reducing participant attrition, or measure suicide fatalities such that missingness because of fatality may be investigated. Therefore, research should continue to explore this area with triangulation from a range of methodologies. Qualitative research [50, 51], studies using large objective datasets [52], studies that determine co‐predictors of suicide attempts among gamblers [53] and psychological autopsy studies [49] can all for example add important unique strands of evidence.

In conclusion, the present study found evidence of positive associations between harmful gambling and current and future suicide attempts among young UK adults, which was unlikely to be confounded by pre‐existing suicidality. These findings can inform policy stakeholders when considering potential gambling harm prevention measures.

## AUTHOR CONTRIBUTIONS

**Oliver Bastiani:** Data curation; formal analysis; investigation; project administration; resources; software; validation; visualization; writing—original draft; writing—review and editing. **Jasmine Khouja:** Supervision; validation; writing—review and editing. **Anya Skatova:** Project administration; resources; supervision; validation; writing—review and editing. **Philip Newall:** Conceptualization; formal analysis; investigation; methodology; project administration; resources; software; supervision; validation; visualization; writing—original draft; writing—review and editing.

## DECLARATION OF INTERESTS

O.B., J.K. and A.S. declare no competing interests. P.N. is a member of the Advisory Board for Safer Gambling—an advisory group of the Gambling Commission in Great Britain. In the last 3 years, P.N. has contributed to research projects funded by the Academic Forum for the Study of Gambling, Clean Up Gambling, Gambling Research Australia, NSW Responsible Gambling Fund and the Victorian Responsible Gambling Foundation. P.N. has received honoraria for reviewing from the Academic Forum for the Study of Gambling and the Belgium Ministry of Justice, travel and accommodation funding from the Alberta Gambling Research Institute and the Economic and Social Research Institute and open access fee funding from Gambling Research Exchange Ontario.

## Supporting information


**Table S1.** Descriptions of all measured variables.
**Table S2.** Preliminary unadjusted univariate regression results between all variables and past‐year suicide attempts at 24 years.
**Table S3.** Preliminary unadjusted univariate regression results between all variables and past‐year suicide attempts at 25 years.
**Table S4.** Hierarchical adjustments of potential confounding variables for the association between past‐year suicide attempts at 24 years and PGSI scores at 24 years, in the unimputed sample.
**Table S5.** Hierarchical adjustments of potential confounding variables for the association between past‐year suicide attempts at 25 years and PGSI scores at 24 years, in the unimputed (complete‐case) sample.
**Table S6.** Hierarchical adjustments of potential confounding variables for the association between past‐year suicide attempts at 24 years and PGSI scores at 20 years, in the unimputed (complete‐case) sample.
**Table S7.** Hierarchical adjustments of potential confounding variables for the association between past‐year suicide attempts at 25 years and PGSI scores at 20 years, in the unimputed (complete‐case) sample.
**Table S8.** Hierarchical adjustments of potential confounding variables for the association between past‐year suicide attempts at 24 years and categorical changes in PGSI scores from 20 to 24 years, in the unimputed (complete‐case) sample.
**Table S9.** The number and percentage of participants for whom missing data was imputed for each variable.

## Data Availability

Due to ALSPAC policy, we cannot share our datasets ourselves. However, if others wish to extract the same data as used in this study, they can apply for access via: https://www.bristol.ac.uk/alspac/researchers/access/.
