# Supplementary material for: The relationship between Problem Gambling Severity Index scores and suicidality: Results of a 9‐year cohort study of young United Kingdom adults
Source: Addiction. 2025 Sep 16;121(1):196–207. doi: 10.1111/add.70156 (PMC12710842; doi:10.1111/add.70156)
Supplement: Supplementary file 1 — Table S1. Descriptions of all measured variables. Table S2. Preliminary unadjusted univariate regression results between all variables and past‐year suicide attempts at 24 years. Table S3. Preliminary unadjusted univariate regression results between all variables and past‐year suicide attempts at 25 years. Table S4. Hierarchical adjustments of potential confounding variables for the association between past‐year suicide attempts at 24 years and PGSI scores at 24 years, in the unimputed sample. Table S5. Hierarchical adjustments of potential confounding variables for the association between past‐year suicide attempts at 25 years and PGSI scores at 24 years, in the unimputed (complete‐case) sample. Table S6. Hierarchical adjustments of potential confounding variables for the association between past‐year suicide attempts at 24 years and PGSI scores at 20 years, in the unimputed (complete‐case) sample. Table S7. Hierarchical adjustments of potential confounding variables for the association between past‐year suicide attempts at 25 years and PGSI scores at 20 years, in the unimputed (complete‐case) sample. Table S8. Hierarchical adjustments of potential confounding variables for the association between past‐year suicide attempts at 24 years and categorical changes in PGSI scores from 20 to 24 years, in the unimputed (complete‐case) sample. Table S9. The number and percentage of participants for whom missing data was imputed for each variable. [file ADD-121-196-s001.docx]

Supplementary Table 1. Descriptions of all measured variables

Supplementary Table 2. Preliminary unadjusted univariate regression results between all variables and past-year suicide attempts at 24 years

Supplementary Table 3. Preliminary unadjusted univariate regression results between all variables and past-year suicide attempts at 25 years

Supplementary Table 4. Hierarchical adjustments of potential confounding variables for the association between past-year suicide attempts at 24 years and PGSI scores at 24 years, in the unimputed sample

Supplementary Table 5. Hierarchical adjustments of potential confounding variables for the association between past-year suicide attempts at 25 years and PGSI scores at 24 years, in the unimputed (complete-case) sample.

Supplementary Table 6. Hierarchical adjustments of potential confounding variables for the association between past-year suicide attempts at 24 years and PGSI scores at 20 years, in the unimputed (complete-case) sample.

Supplementary Table 7. Hierarchical adjustments of potential confounding variables for the association between past-year suicide attempts at 25 years and PGSI scores at 20 years, in the unimputed (complete-case) sample.

Supplementary Table 8. Hierarchical adjustments of potential confounding variables for the association between past-year suicide attempts at 24 years and categorical changes in PGSI scores from 20 to 24 years, in the unimputed (complete-case) sample.

Supplementary Table 9. The number and percentage of participants for whom missing data was imputed for each variable.

Supplementary Table 1. Descriptions of all measured variables

| Variable | Measurement | Age | Dates Collected | Response  Rates  (total sample = 15,645, at birth) |
| --- | --- | --- | --- | --- |
| Lifetime Suicide Attempt Prevalence | (If participants answered that they had ever hurt themselves in any way)  “On any of the occasions when you have hurt yourself on purpose, have you ever seriously wanted to kill yourself?”, with answers: (Yes/No).  Responded “I wanted to die” as a reason for self harm. Answers from both questions pooled to indicate lifetime suicidality cases vs non-cases. | 16 Years | October 2007 – August 2009 | 5,118 |
| Past-Year Suicide Attempt Prevalence | “Have any of these happened since you were 23 years old and did they affect you? – You attempted suicide”, with answers: (“No, did not happed”, “Yes, but didn’t affect me at all”, “Yes, mildly affected”, “Yes, moderately affected”, “Yes, affected me a lot”). Coded as a past-year suicide attempt if responded “yes” (regardless of affect).  (If participants answered “yes” to lifetime self-harm and “yes” to seriously wanting to kill yourself) “If yes, when was the last time you hurt yourself on purpose and you seriously wanted to kill yourself?”, with answers: (“In the last week”, “More than a week ago, but in the last year”, “More than a year ago”). Responses that indicated past-year suicide attempts were coded as cases vs non-cases (had not self-harmed, or had wanted to kill themselves but not in the past year).  Cases of past-year suicide attempts were pooled from both questions to create an overall prevalence of past-year suicide attempts (prevalence coded as Yes/No). | 24 Years | November 2016 – August 2017 | 4,331 |
| Past-Year Suicide Attempt Prevalence | “Have any of these happened in the past 12 months and did they affect you? – You attempted suicide”, with answers: (“No, did not happed”, “Yes, but didn’t affect me at all”, “Yes, mildly affected”, “Yes, moderately affected”, “Yes, affected me a lot”). Coded as a past-year suicide attempt if responded “yes” (regardless of affect). | 25 Years | November 2017 – July 2018 | 4,073 |
| Past-Year Gambling Prevalence | 17-Item questionnaire, derived from the British Gambling Prevalence Survey (BGPS). 13 of the 17 item were used. This asked participants of their past-12 month frequency of the following gambling formats:  1. National Lottery (excluding scratchcards)  2. Scratchcards (excluding newspaper or magazine scratchcards)  3. Football pools (excluding betting on football matches with bookmaker)  4. Bingo cards or tickets (including bingo hall, excluding online bingo)  5. Fruit slot machines (excluding quiz machines)  6. Virtual gaming machines in bookmakers to bet on virtual roulette, keno, bingo, etc. (excluding quiz machines)  7. Table games (roulette, dice or cards) in a casino (excluding poker or casino games played online)  8. Online gambling like poker, bingo, slot-machine-style games, casino games 'for money' (inc. computer, mobile phone, interactive TV, not inc. online bookmakers or betting exchanges)  9. Online betting with bookmaker (inc. through computer, mobile phone or interactive TV, not inc. bets made with a betting exchange or spread-betting)  10. Betting exchange ("peer-to-peer" betting)  11. Bet on horse races in a bookmaker's, by phone, or at the track (inc. tote betting and virtual horse races shown in a bookmaker's, not inc. online bookmakers or betting exchanges)  12. Spread-betting  13. Bet privately, playing cards or games for money with friends, family or colleagues  Four response options were possible, ranging from “Every day”, to “ Not within the past 12 months”. These were coded as past-year occurrence of gambling participation in any form vs non. | 24 Years | November 2016 – August 2017 | 4,215 |
| Problem Gambling Severity | The Problem Gambling Severity Index (PGSI) asked participant 9 questions on their gambling behaviour in the past 12 months. Their were four responses possible for each question, ranging from “Never” (Coded as 0) to “Almost always” (Coded as 3). Total scores ranged from 0 to 27, with higher scores indicating a greater likelihood of problem gambling. | 20 Years | September 2012 – July 2013 | 2,622 |
| Problem Gambling Severity | The Problem Gambling Severity Index (PGSI) asked participant 9 questions on their gambling behaviour in the past 12 months. Their were four responses possible for each question, ranging from “Never” (Coded as 0) to “Almost always” (Coded as 3). Total scores ranged from 0 to 27, with higher scores indicating a greater likelihood of problem gambling. | 24 Years | November 2016 – August 2017 | 1,716 |
| Changes in Problem Gambling Severity | Participants total PGSI scores at 20 years were subtracted from participants total PGSI scores at 24 years, to indicate the numerical change in PGSI scores for each participant. Following Wardle et.al. (2023) methods, these were coded: No change / Increase of 1 PGSI score or more / Decrease of 1 PGSI score or more. | 20 – 24 Years | September 2012 – August 2017 | 893 |
| Parental Social Class | Highest OPCS job code, response options of:  1: I  2: II  3: III (non-manual)  4: III (manual)  5: IV  6: V  For each parent, coded to:  0: Non-manual: professional, managerial, or skilled professions  1: Manual: party or unskilled occupations  These were combined to generate the presence of a skilled parent (vs non), if a child had either a non-manual mother or father. | 32 Weeks Gestation | March 1991 – Jan 1993 | 11,550 |
| Maternal Education | Participant’s mothers were asked to indicate their education level from:  1: CSE/none  2: Vocational  3: O-level  4: A-level  5: Degree  This was coded to having achieved A-level or Degree vs. O-level/Vocational/CSE/Non. | 32 Weeks Gestation | March 1991 – Jan 1993 | 12,469 |
| Crowding Index | The number of people living in the household, divided by the number of rooms in the household (excluding kitchens and bathrooms). Higher scores indicated greater household crowding.  A binary variable was created, comparing those with an index of >1 to 1>. | 8 Weeks Gestation | September 1990 – October 1992 | 13,680 |
| Index of Multiple Deprivation | The Index of Multiple Deprivation (IMD) accounts for census data from 7 socioeconomic domains (income, employment, health and disability, education, skills and training, barriers to housing and services, living environment, and crime). Higher scores indicate greater social deprivation. | 20 years | 2010 | 8,101 |
| Home Ownership Status | Mothers were asked to indicate their home ownership status during pregnancy from:  0. being bought/mortgaged  1. owned – with no mortgage to pay  2. rented from council  3. rented from private landlord (furnished)  4. rented from private landlord (unfurnished)  5. rented from housing association  Re-coded as mortgaged/owned vs. rented. | 8 Weeks Gestation | September 1990 – October 1992 | 13,477 |
| Economic Activity | A questionnaire asked participants were asked “If they were currently in employment or doing and education or training”: with responses (Yes/No) | 20 Years | September 2012 – July 2013 | 4,139 |
| Economic Activity | Clinicians asked participants about their current education/employment status. Participants were coded as economically active if they answered “Yes” to the following:   - Full-time education - Part-time education - Full-time employment - Part-time employment - In a training scheme   If participants answered “No” to all of these, they were coded as not being economically active. | 24 Years | Clinic – 24 Years | 3,883 |
| Sex | Participant’s sex assigned at birth. Coded as Male/Female | Birth | N/A | 15,030 |
| Hyperactivity | The Strengths and Difficulties Questionnaire (SDQ) was used to measure hyperactivity. For each SDQ subscale, possible scores ranged from 0 to 10. Scores were binary coded, with cut-offs for ‘abnormal’ scoring (reflected 10% of the population, different for each subscale). For hyperactivity scores of 8 or above were considered as ‘abnormal’. | 9.5 years (115 months) | February 2001 – November 2002 | 8,140 |
| Conduct Problems | The SDQ was used to measure conduct problems. For each SDQ subscale, possible scores ranged from 0 to 10. Scores were binary coded, with cut-offs for ‘abnormal’ scoring (reflected 10% of the population, different for each subscale). For hyperactivity scores of 4 or above were considered as ‘abnormal’. | 9.5 Years (115 months) | February 2001 – November 2002 | 8,160 |
| Locus of Control | Participants completed the 12-item Nowicki-Strickland Locus of Control Scale. Items could be answered with Yes/No, and scored 1 to 0, respectively. Scores for all 12 items were summed to create an overall locus of control score. Greater scores indicate a greater likelihood of an external locus of control. | 16 Years (198 months) | October 2007 – August 2009 | 4,766 |
| Regular social Media Use | Participants were asked “Do you have a page or profile on these sites or apps and how often do you use them?”, with 21 different social media options to respond to. For each social media, participants had 6 response options, each indicating a frequency of use, ranging from “No” to “Yes, use less often (than monthly)”. Regular social media use was coded as participants having used at least one of form of social media weekly vs not using any/ not using any using non-weekly. | 24 Years | November 2016 – August 2017 | 4,191 |
| Attention Deficit Hyperactivity Disorder (ADHD) Diagnosis | Parent’s completed a child-based questionnaire, which asked whether their child had a clinical diagnosis of any ADHD disorder (DSM-IV). This was coded as cases of any ADHD diagnoses vs. non. | 7.5 Years (91 months) | July 1998 – October 2000 | 8,198 |
| Problem Drinking Likelihood | Participants completed the 3-item Alcohol Use Disorders Identification Test Short Form (AUDIT-C). Scores ranged from 0-12, and were used as a continuous measure, with higher scores indicating a greater likelihood of the participant having an alcohol use disorder. | 24 Years | N/A | 3,927 |
| Smoking Status | (If participants had already answered “Yes” to having ever smoked a whole cigarette AND “Yes” to having smoked any cigarettes in the past 30 days) Participants were asked if they smoke every week (Yes/No) | 24 Years | November 2016 – August 2017 | 4,340 |
| Maternal Gambling | Mothers completed the South Oaks Gambling Screen (SOGS). This assessed the frequency of their gambling across 11 different forms of gambling, with 4 response options ranging from “Once a week or more” to “Rarely or not at all”. Cases of maternal gambling were coded if participants’ mothers indicated that they participated in any form of gambling weekly or more (vs not participating in any forms of gambling weekly or more frequently). | 6 Years (73 months) | February 1997 – March 1999 | 8,205 |
| Parental Gambling | Participants fathers also completed the SOGS questionnaire, and we coded their responses in the same way as participants mothers. Parental gambling was measured as the prevalence of participants parents having gambled “Nowadays” weekly or more frequently vs. non. | 6 Years (73 months) | February 1997 – March 1999 | 10,919 |

Supplementary Table 2. Preliminary unadjusted univariate regression results between all variables and past-year suicide attempts at 24 years

| Univariate Logistic Regression Results with Past-Year Suicide Attempts at 24 years (ALL CASES) | | | | | | |
| --- | --- | --- | --- | --- | --- | --- |
| Variable | OR | Std. err | z | P>\|z\| | 95% CI | N. obs |
| PGSI 20 | 1.182 | .065 | 3.01 | 0.003 | 1.06 - 1.31 | 1,759 |
| PGSI 24 | 1.129 | .038 | 3.57 | 0.000 | 1.05 - 1.20 | 1,715 |
| PGSI Change 20-24 (Categorical) | 1.538 | .384 | 1.72 | 0.085 | .942 - 2.51 | 892 |
| Parental Social Class | 1.459 | .419 | 1.31 | 0.189 | .830 - 2.56 | 3,715 |
| Maternal Education | 1.241 | .161 | 1.67 | 0.096 | .962 - 1.60 | 3,874 |
| Crowding Index | 1.242 | .151 | 1.78 | 0.075 | .978 - 1.57 | 3,935 |
| IMD in 2010 | 1.059 | .097 | 0.63 | 0.531 | .884 - 1.26 | 2,339 |
| Home Ownership | 1.444 | .368 | 1.44 | 0.150 | .876 - 2.38 | 3,882 |
| Edu/Employ 20yrs | .3872 | .130 | -2.81 | 0.005 | .200 - .749 | 2,847 |
| Edu/Employ 24yrs | .2402 | .069 | -4.95 | 0.000 | .136 - .422 | 3,021 |
| Hyperactivity | 2.513 | .752 | 3.08 | 0.002 | 1.39 - 4.52 | 3,484 |
| Conduct Problems | 2.194 | .622 | 2.77 | 0.006 | 1.25 - 3.82 | 3,481 |
| Locus of Control | 1.207 | .066 | 3.44 | 0.001 | 1.08 - 1.34 | 2,886 |
| Social Media | 1.345 | .256 | 1.56 | 0.120 | .925 - 1.95 | 4,187 |
| ADHD Diagnosis | 1.992 | 1.46 | 0.94 | 0.347 | .473 - 8.38 | 3,349 |
| AUDIT 24yrs | .9936 | .048 | -0.13 | 0.896 | .903 - 1.09 | 3,047 |
| Smoke 24yrs | 1.400 | .555 | 0.85 | 0.397 | .643 - 3.04 | 4,331 |
| Maternal Gambling | .6713 | .161 | -1.65 | 0.098 | .418 - 1.07 | 3,191 |
| Parental Gambling | .8201 | .172 | -0.94 | 0.345 | .543 - 1.23 | 3,571 |

Supplementary Table 3. Preliminary unadjusted univariate regression results between all variables and past-year suicide attempts at 25 years

| Univariate Logistic Regression Results with Past-Year Suicide Attempts at 25 years | | | | | | |
| --- | --- | --- | --- | --- | --- | --- |
| Variable | OR | Std. err | z | P>\|z\| | 95% CI | N. obs |
| PGSI 20 | 1.156 | .088 | 1.90 | 0.057 | .995 - 1.34 | 1,694 |
| PGSI 24 | 1.175 | .051 | 3.68 | 0.000 | 1.07 - 1.28 | 1,316 |
| PGSI Change 20-24 (Categorical) | 2.099 | .826 | 1.88 | 0.060 | .970 - 4.54 | 732 |
| Parental Social Class | 2.320 | .748 | 2.61 | 0.009 | 1.23 - 4.36 | 3,506 |
| Maternal Education | 1.517 | .257 | 2.45 | 0.014 | 1.08 - 2.11 | 3,646 |
| Crowding Index | 1.072 | .219 | 0.34 | 0.731 | .718 - 1.60 | 3,719 |
| IMD in 2010 | 1.108 | .139 | 0.82 | 0.413 | .866 - 1.41 | 2,182 |
| Home Ownership | 1.603 | .512 | 1.48 | 0.140 | .857 - 3.00 | 3,669 |
| Edu/Employ 20yrs | .1656 | .058 | -5.09 | 0.000 | .082 - .331 | 2,741 |
| Edu/Employ 24yrs | .1584 | .053 | -5.49 | 0.000 | .082 - .305 | 2,793 |
| Hyperactivity | 2.121 | .876 | 1.82 | 0.069 | .943 - 4.76 | 3,263 |
| Conduct Problems | 2.796 | 1.01 | 2.83 | 0.005 | 1.37 - 5.70 | 3,271 |
| Locus of Control | 1.149 | .087 | 1.82 | 0.069 | .989 - 1.33 | 2,775 |
| Social Media | 1.736 | .495 | 1.93 | 0.053 | .992 - 3.03 | 3,272 |
| ADHD Diagnosis | 4.868 | 3.63 | 2.12 | 0.034 | 1.12 - 21.0 | 3,168 |
| AUDIT 24yrs | .8569 | .053 | -2.47 | 0.014 | .758 - .968 | 2,808 |
| Smoke 24yrs | 1.527 | .918 | 0.70 | 0.481 | .469 - 4.96 | 3,360 |
| Maternal Gambling | .7285 | .225 | -1.02 | 0.307 | .396 - 1.33 | 3,035 |
| Parental Gambling | .7375 | .205 | -1.09 | 0.275 | .426 - 1.27 | 3,387 |

Supplementary Table 4. Hierarchical adjustments of potential confounding variables for the association between past-year suicide attempts at 24 years and PGSI scores at 24 years, in the unimputed (complete-case) sample.

|  |  | Model 1  (*n* = 1,715, *case n* = 38) | Model 2  (*n* = 1,080, *case n* = 18) | Model 3  (*n* = 861, *case n* = 13) | Model 4  (*n* = 710, *case n* = 11) |  |
| --- | --- | --- | --- | --- | --- | --- |
| PGSI Scores at 24 years | |  |  |  |  |  |
|  | Odds Ratio  (95% Confidence intervals) | 1.13 (1.06 – 1.21) | 1.11 (1.01 – 1.22) | 1.05 (0.91 - 1.21) | 1.12 (0.97 – 1.29) |  |
|  | p-value | < .001 | .030 | .528 | .137 |  |
| Sex | |  |  |  |  |  |
|  | Odds Ratio  (95% Confidence intervals) |  | 1.09 (0.41 – 2.92) | 0.98 (0.30 – 3.15) | 1.56 (0.41 – 5.89) |  |
|  | p-value |  | .859 | .968 | .513 |  |
| Economic Activity | |  |  |  |  |  |
|  | Odds Ratio  (95% Confidence intervals) |  | 0.17 (0.06 – 0.51) | 0.10 (0.03 – 0.32) | 0.11 (0.03 – 0.39) |  |
|  | p-value |  | .001 | < .001 | .001 |  |
| Maternal Education | |  |  |  |  |  |
|  | Odds Ratio  (95% Confidence intervals) |  | 0.52 (0.19 – 1.44) | 0.60 (0.19 – 1.94) | 0.80 (0.23 – 2.83) |  |
|  | p-value |  | .207 | .395 | .732 |  |
| Hyperactivity | |  |  |  |  |  |
|  | Odds Ratio  (95% Confidence intervals) |  |  | omitted | omitted |  |
|  | p-value |  |  |  |  |  |
| AUDIT scores | |  |  |  |  |  |
|  | Odds Ratio  (95% Confidence intervals) |  |  | 0.96 (0.75 – 1.22) | 1.03 (0.79 – 1.35) |  |
|  | p-value |  |  | .725 | .812 |  |
| Lifetime Suicide Attempt Prevalence at 16 years | |  |  |  |  |  |
|  | Odds Ratio  (95% Confidence intervals) |  |  |  | 2.02 (0.21 – 19.25) |  |
|  | p-value |  |  |  | .543 |  |

Supplementary Table 5. Hierarchical adjustments of potential confounding variables for the association between past-year suicide attempts at 25 years and PGSI scores at 24 years, in the unimputed (complete-case) sample.

|  | | Model 1  (*n* = 1,316, *case n* = 18) | Model 2  (*n* = 891, *case n* = 11) | Model 3  (*n* = 740, *case n* = 10) | Model 4  (*n* = 625, *case n* = 7) |  |
| --- | --- | --- | --- | --- | --- | --- |
| PGSI Scores at 24 years | |  |  |  |  |  |
|  | Odds Ratio  (95% Confidence intervals) | 1.18 (1.08 – 1.28) | 1.10 (0.98 – 1.24) | 1.09 (0.97 – 1.23) | 1.29 (1.11 – 1.51) |  |
|  | p-value | < .001 | .096 | .170 | .001 |  |
| Sex | |  |  |  |  |  |
|  | Odds Ratio  (95% Confidence intervals) |  | 0.58 (0.17 – 2.04) | 0.37 (0.09 – 1.54) | 0.09 (0.01 – 0.86) |  |
|  | p-value |  | .398 | .170 | .037 |  |
| Economic Activity | |  |  |  |  |  |
|  | Odds Ratio  (95% Confidence intervals) |  | 0.12 (0.03 – 0.46) | 0.14 (0.03 – 0.57) | 0.14 (0.02 – 0.83) |  |
|  | p-value |  | .002 | .006 | .031 |  |
| Maternal Education | |  |  |  |  |  |
|  | Odds Ratio  (95% Confidence intervals) |  | 0.21 (0.04 – 1.04) | 0.24 (0.05 – 1.25) | 0.09 (0.01 – 1.12) |  |
|  | p-value |  | .056 | .089 | .061 |  |
| Hyperactivity | |  |  |  |  |  |
|  | Odds Ratio  (95% Confidence intervals) |  |  | 6.75 (1.07 – 42.8) | 5.87 (0.39 – 87.9) |  |
|  | p-value |  |  | .043 | .200 |  |
| AUDIT scores | |  |  |  |  |  |
|  | Odds Ratio  (95% Confidence intervals) |  |  | 0.74 (0.56 – 0.98) | 0.71 (0.49 – 1.02) |  |
|  | p-value |  |  | .036 | .064 |  |
| Lifetime Suicide Attempt Prevalence at 16 years | |  |  |  |  |  |
|  | Odds Ratio  (95% Confidence intervals) |  |  |  | 2.57 (0.14 – 45.6) |  |
|  | p-value |  |  |  | .520 |  |

Supplementary Table 6. Hierarchical adjustments of potential confounding variables for the association between past-year suicide attempts at 24 years and PGSI scores at 20 years, in the unimputed (complete-case) sample.

|  | | Model 1  (*n* = 1,759, *case n* = 42) | Model 2  (*n* = 1,225, *case n* = 26) | Model 3  (*n* = 992, *case n* = 22) | Model 4  (*n* = 887, *case n* = 19) |  |
| --- | --- | --- | --- | --- | --- | --- |
| PGSI Scores at 20 years | |  |  |  |  |  |
|  | Odds Ratio  (95% Confidence intervals)0. | 1.18 (1.06 - 1.32) | 1.26 (1.11 – 1.43) | 1.26 (1.11 – 1.45) | 1.28 (1.10 – 1.49) |  |
|  | p-value | .003 | < .001 | .001 | .001 |  |
| Sex | |  |  |  |  |  |
|  | Odds Ratio  (95% Confidence intervals) |  | 2.92 (1.06 – 8.04) | 2.50 (0.87 – 7.20) | 2.33 (0.72 – 7.52) |  |
|  | p-value |  | .039 | .090 | .159 |  |
| Economic Activity | |  |  |  |  |  |
|  | Odds Ratio  (95% Confidence intervals) |  | 0.19 (0.07 – 0.50) | 0.19 (0.07 – 0.55) | 0.21 (0.06 – 0.71) |  |
|  | p-value |  | .001 | .002 | .012 |  |
| Maternal Education | |  |  |  |  |  |
|  | Odds Ratio  (95% Confidence intervals) |  | 0.69 (0.31 - ) | 0.81 (0.33 – 1.96) | 0.79 (0.30 – 2.09) |  |
|  | p-value |  | 1.57 | .634 | .635 |  |
| Hyperactivity | |  | .377 |  |  |  |
|  | Odds Ratio  (95% Confidence intervals) |  |  | omitted | omitted |  |
|  | p-value |  |  |  |  |  |
| AUDIT scores | |  |  |  |  |  |
|  | Odds Ratio  (95% Confidence intervals) |  |  | 1.05 (0.88 – 1.26) | 1.12 (0.92 – 1.37) |  |
|  | p-value |  |  | .586 | .255 |  |
| Lifetime Suicide Attempt Prevalence at 16 years | |  |  |  |  |  |
|  | Odds Ratio  (95% Confidence intervals) |  |  |  | 4.76 (1.43 – 15.8) |  |
|  | p-value |  |  |  | .011 |  |

Supplementary Table 7 Hierarchical adjustments of potential confounding variables for the association between past-year suicide attempts at 25 years and PGSI scores at 20 years, in the unimputed (complete-case) sample.

|  | | Model 1  (*n* = 1,694, *case n* = 23) | Model 2  (*n* = 1,161, *case n* = 15) | Model 3  (*n* = 943, *case n* = 13) | Model 4  (*n* = 847, *case n* = 11) |
| --- | --- | --- | --- | --- | --- |
| PGSI Scores at 20 years | |  |  |  |  |
|  | Odds Ratio  (95% Confidence intervals) | 1.16 (1.00 – 1.34) | 1.11 (0.88 – 1.41) | 1.12 (0.89 – 1.39) | 1.24 (0.95 – 1.62) |
|  | p-value | .057 | .365 | .333 | .112 |
| Sex | |  |  |  |  |
|  | Odds Ratio  (95% Confidence intervals) |  | 0.64 (0.22 – 1.86) | 0.47 (0.15 - 1.49) | 0.39 (0.11 – 1.39) |
|  | p-value |  | .408 | .197 | .146 |
| Economic Activity | |  |  |  |  |
|  | Odds Ratio  (95% Confidence intervals) |  | 0.12 (0.04 – 0.37) | 0.13 (0.04 – 0.47) | 0.15 (0.04 – 0.62) |
|  | p-value |  | < .001 | .002 | .009 |
| Maternal Education | |  |  |  |  |
|  | Odds Ratio  (95% Confidence intervals) |  | 0.13 (0.03 – 0.61) | 0.17 (0.04 – 0.81) | 0.19 (0.04 – 0.95) |
|  | p-value |  | .009 | .026 | .043 |
| Hyperactivity | |  |  |  |  |
|  | Odds Ratio  (95% Confidence intervals) |  |  | omitted | omitted |
|  | p-value |  |  |  |  |
| AUDIT scores | |  |  |  |  |
|  | Odds Ratio  (95% Confidence intervals) |  |  | 0.89 (0.71 – 1.11) | 0.85 (0.66 – 1.10) |
|  | p-value |  |  | .304 | .214 |
| Lifetime Suicide Attempt Prevalence at 16 years | |  |  |  |  |
|  | Odds Ratio  (95% Confidence intervals) |  |  |  | 2.34 (0.26 – 21.0) |
|  | p-value |  |  |  | .448 |

| Supplementary Table 8. Hierarchical adjustments of potential confounding variables for the association between past-year suicide attempts at 24 years and categorical changes in PGSI scores from 20 to 24 years, in the unimputed (complete-case) sample. | | | | | | | |
| --- | --- | --- | --- | --- | --- | --- | --- |
| Variable | | Model 1 (*n* = 892, *case n* = 20) | Model 2 (*n* = 892, *case n* = 20) | Model 3 (*n* = 614, *case n* = 12) | Model 4 (*n* = 502, *case n* = 10) | Model 5 (*n* = 448, *case n* = 9) |  |
| PGSI Score Change 20-24 Years (Categorical) | |  |  |  |  |  |  |
|  | PGSI score increased  Odds Ratio  (95% Confidence intervals) | 1.90 (0.58 – 6.28) | 1.60 (0.46 – 5.54) | 0.43 (0.04 – 4.63) | 0.50 (0.04 – 5.80) | 0.62 (0.05 – 7.02) |  |
|  | p-value | .290 | .454 | .489 | .578 | .700 |  |
|  | PGSI score decreased  Odds Ratio  (95% Confidence intervals) | 2.34 (0.86 – 6.38 | 1.75 (0.97 – 1.30) | 1.35 ( 0.59 – 9.41) | 2.70 (0.58 – 12.58) | 1.78 (0.33 – 9.50) |  |
|  | p-value | .096 | .320 | .226 | .207 | .502 |  |
| PGSI Scores at 20 years | |  |  |  |  |  |  |
|  | Odds Ratio  (95% Confidence intervals) |  | 1.12 (0.97 – 1.30) | 1.23 (1.03 – 1.46) | 1.22 (1.02 – 1.47) | 1.26 (1.04 – 1.53) |  |
|  | p-value |  | .127 | .021 | .031 | .020 |  |
| Sex | |  |  |  |  |  |  |
|  | Odds Ratio  (95% Confidence intervals) |  |  | 2.34 (0.62 – 8.85) | 1.85 (0.44 – 7.78) | 2.24 (0.48 – 10.51) |  |
|  | p-value |  |  | .210 | 0.401 | .307 |  |
| Economic Activity | |  |  |  |  |  |  |
|  | Odds Ratio  (95% Confidence intervals) |  |  | 0.16 (0.04 – 0.66) | 0.12 (0.03 – 0.56) | 0.17 (0.03 – 0.98) |  |
|  | p-value |  |  | .012 | .007 | .047 |  |
| Maternal Education | |  |  |  |  |  |  |
|  | Odds Ratio  (95% Confidence intervals) |  |  | 0.45 (0.13 – 1.57) | 0.62 (0.15 – 2.52) | 0.71 (0.17 – 3.01) |  |
|  | p-value |  |  | .208 | .501 | .643 |  |
| Hyperactivity | |  |  |  |  |  |  |
|  | Odds Ratio  (95% Confidence intervals) |  |  | 0.05 (0.01 – 0.29) | omitted | omitted |  |
|  | p-value |  |  | .001 |  |  |  |
| AUDIT scores | |  |  |  |  |  |  |
|  | Odds Ratio  (95% Confidence intervals) |  |  |  | 0.93 (0.70 – 1.23) | 0.99 (0.71 – 1.36) |  |
|  | p-value |  |  |  | .595 | .928 |  |
| Lifetime Suicide Attempt Prevalence at 16 years | |  |  |  |  |  |  |
|  | Odds Ratio  (95% Confidence intervals) |  |  |  |  | 2.64 (0.29 – 24.47) |  |
|  | p-value |  |  |  |  | .392 |  |

| Supplementary Table 9. The number and percentage of participants for whom missing data was imputed for each variable. | | |
| --- | --- | --- |
| Variable | Imputed N | Imputed % |
| Suicide attempts at 16 years | 888 | 31.7% |
| Suicide attempts at 24 years | 0 | 0.00% |
| Suicide attempts at 25 years | 658 | 23.5% |
| PGSI Scores at 20 years | 1,467 | 52.4% |
| PGSI Scores at 24 years | 1,086 | 57.5% |
| PGSI Scores change between 20-24 years | 1,909 | 68.2% |
| Sex | 0 | 0.00% |
| Economic Activity at 24 years | 894 | 31.9% |
| Maternal Education | 302 | 10.8% |
| Hyperactivity | 555 | 19.8% |
| AUDIT scores | 881 | 31.5% |
| * All samples are out of the total possible N=2,801, which reflects the overall ALSPAC sample which has data for past-year suicide attempts and British Gambling Prevalence Survey (BGPS) scores > 0 (which is a prerequisite of conducting the Problem Gambling Severity Index, PGSI). | | |
